# Age- and severity-adjusted treatment of proximal humerus fractures in children and adolescents—A systematical review and meta-analysis

**DOI:** 10.1371/journal.pone.0183157

**Published:** 2017-08-24

**Authors:** Lisa Hohloch, Helge Eberbach, Ferdinand C. Wagner, Peter C. Strohm, Kilian Reising, Norbert P. Südkamp, Jörn Zwingmann

**Affiliations:** 1 Department of Orthopedics and Trauma Surgery, Medical Center—Albert-Ludwigs-University of Freiburg, Faculty of Medicine, Albert-Ludwigs-University of Freiburg, Freiburg, Germany; 2 Clinic for Orthopedics and Trauma Surgery, Klinikum Bamberg, Bamberg, Germany; Klinikum rechts der Isar der Technischen Universitat Munchen, GERMANY

## Abstract

**Background:**

Fractures of the proximal humerus in patients under the age of 18 years show a low incidence; existing clinical studies only comprise small patient numbers. Different treatment methods are mentioned in the literature but a comparison of the outcome of these methods is rarely made. Up to now, no evidence-based algorithm for conservative and operative treatment is available. The aim of this systematic review with meta-analysis was therefore to gather the best evidence of different treatment methods and their associated functional outcome, complication rates, rates of limb length discrepancies and radiological outcome.

**Methods and findings:**

The OVID database was systematically searched on September 30th in 2016 in order to find all published clinical studies on the subject of proximal humerus fractures of patients ≤18 years. Exclusion criteria were previously defined. The Coleman Methodology Score was used to evaluate the quality of the single studies. 886 studies have been identified by the search strategy. 19 studies with a total of 643 children (mean age: 11.8 years) were included into the meta-analysis with a mean Coleman Methodology Score of 71 ± 7.4 points. 18 of the 19 studies eligible for inclusion were retrospective ones, of the best quality available (mean follow-up ≥ 1 year, mean follow-up rate ≥ 65%). 56% of the patients were male. Proximal humerus fractures were treated conservatively in 41% and surgically in 59% of the cases (Elastic Stable Intramedullary Nailing (ESIN): 31%; K-wires: 20%; 8% other methods, e.g. plate osteosynthesis, olecranon traction). The overall success rate (good/excellent outcome) for all treatment methods was 93%. The success rate of ESIN (98%) and of K- wire fixation (95%) was significantly higher (p = 0.01) than the success rate of conservative treatment options (91%). A subgroup analysis of severely displaced fractures (Neer grade III/IV, angulation ≥ 20°) resulted in a change of success rates, to the disadvantage of conservative treatment methods (conservative treatment 82%, ESIN 98%, K-wires 95%; p < 0.001). Complication rates did not differ to a significant extent. 9% of the complications occurred in the patients treated by K-wire fixation, 8% if a conservative treatment option was chosen and 7% in the fractures that were stabilized by ESIN. A change from a one-nail technique to a two-nail technique reduced the complication rate of ESIN significantly. Follow-up X- rays without residual deformity could be found in 96% of the patients treated by ESIN, a rate which was higher than in the patients treated conservatively (93%) or by K-wire fixation (88%). The rate of arm length discrepancies at final follow- up was lower if the fractures were stabilized by ESIN (4%) than if they were treated conservatively (9%) or by K-wires (19%). An evaluation of age-dependent treatment options was performed.

**Conclusions:**

By performing this meta-analysis an evidence-based treatment algorithm could be introduced to treat the fractures according to the severity of displacement and according to the patient's age. For severely displaced fractures ESIN is the method of choice, with the best clinical and radiological outcome.

## Introduction

Fractures of the proximal humerus in children and adolescents are fractures with a low incidence of 6.8 fractures/10000 children per year with a major proportion of those fractures occurring in the age of 11–15 years.[1]

Until today, no consensus and no evidence-based guideline have been published concerning treatment options in dependence of patient age, fracture severity and grade of displacement.

The high remodelling potential of the physis of the proximal humerus was expected to correct axial deviations and arm length discrepancies especially in younger children as it seems to be responsible for about 80% of the longitudinal growth potential of the humerus.[2,3]

Fractures without or with slight displacement (mainly Neer-Horowitz Grade I or II)[3] were mainly treated conservatively (sling, Desault or Velpeau bandage, cast or hanging cast).[1,3,4] Yet, treatment of significantly displaced fractures has been the subject of a heated discussion since the 1960s. In former times traction and immobilization in a cast in statue-of-liberty- or salute-position were used for severely displaced fractures.[3,5] Those treatment options receded into the background due to complications and a much longer hospital stay.[3–5] Recently published studies increasingly focussed on surgical treatment of severely displaced fractures. From the 1980s onwards surgical treatment was mainly performed by K-wires, screws and plates.[6–9] In 1976 Metaizeau invented the surgical method of elastic stable intramedullary nailing (ESIN). A description of a technique to perform an ESIN- osteosynthesis of proximal humeral fractures in children and adolescents was given in the 1980s.[10,11] In the past ten years several studies have been published describing the surgeons’ experience with ESIN.[12–17]

On closer inspection, existing reviews are mainly expert opinions without clear evidence.[4,18,19] Pahlavan et al. came to the conclusion that it was nearly impossible to determine in which cases operative treatment is indicated. They claim that age cut-offs for surgical treatment are only theoretical in nature as skeletal maturity does not necessarily match chronologic age.[18] Bishop et. al. advocate an individualized approach based on age and extent of fracture displacement according to the proposal of Dobbs et al. who developed a treatment algorithm based on a guideline for acceptable positions for fracture alignment.[4,7,20]

In another guideline published by the German society of pediatric surgery recommendations were formulated by an expert group and were based on the patients’ age, the extent of fracture displacement and comorbidities. Limits for a switch to surgical treatment were determined according to the natural limits of a child’s proximal physis to correct deformities on its own, without further reposition or fracture stabilization.[21] Nonetheless, the individual reasons for these correction limits are still unknown.

The aim of this systematic review with meta-analysis was to provide the best possible evidence-based instructions on how to treat fractures of the proximal humerus of children and adolescents. In addition, a detailed analysis of complication rates, growth disorders and radiological anomalies was performed.

## Methods

### Search of the database

A systematic review with meta-analysis was performed by an OVID-based literature search. Relevant clinical studies were extracted of the databases MEDLINE, PreMEDLINE, EBM Reviews, Cochrane Database, CINAHL and EMBASE. The search strategy was consistent with the PRISMA guidelines (Preferred Reporting Items for Systematic Reviews and Meta-Analysis, see S1 File, S2 File).[22] The search was limited to studies published in the time period from the first of January 1960 to the 30th of September 2016.

The chosen systematic strategy was as follows: (1) humeral fractures/ or shoulder fractures (2) humerus/in, su (Injuries, surgery), (3) humeral head/in, su (4) shoulder joint/in,su (5) 1 or 2 or 3 or 4 (6) proximal.tw. (7) subcapital. tw. (8) sub-capital. tw. (9) head.tw. (10) epiphysis.tw. (11) humer* tw. (12) 6 or 7 or 8 or 9 or 10 (13) 11 and 12 (14) 5 and 13 (15) limit 14 to/ ("newborn infant (birth to 1 month)" or "infant (1 to 23 months)" or "preschool child (2 to 5 years)" or "child (6 to 12 years)") (16) limit 14 to "adolescent (13 to 18 years)”(17) 15 or 16. Primary inclusion criteria were all clinical studies about treatment options of proximal humerus fractures in children under the age of 18 years. Primary exclusion criteria were case reports, reviews, radiological studies, biomechanical studies, anatomical studies or studies about surgical techniques.

**Fig 1:**
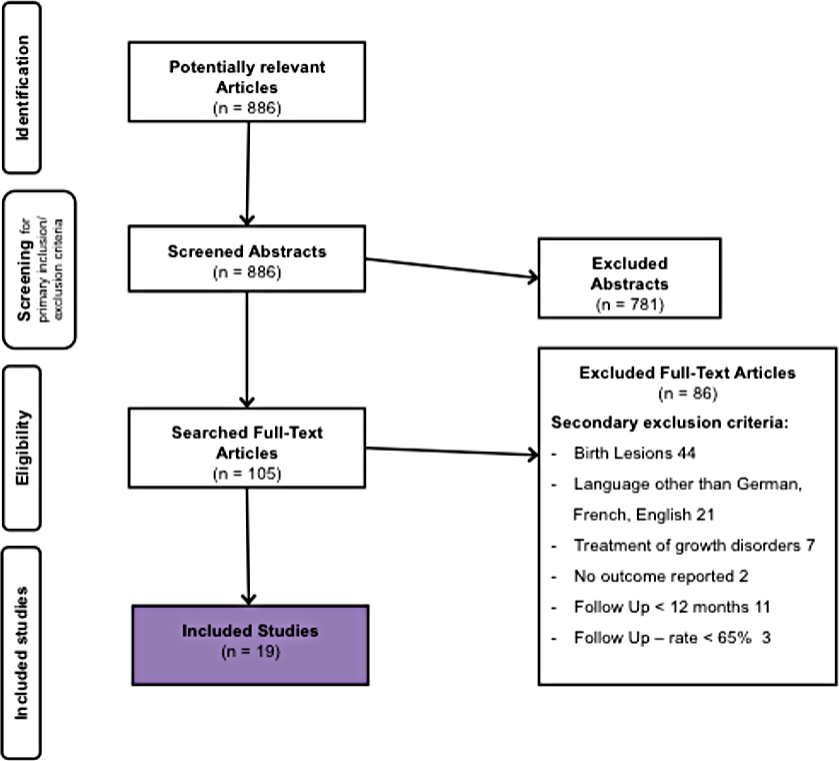
Flow chart of the search strategy and study selection. Criteria for drop-out and subsequent number of studies remaining for further analysis.

Conduction of search, data extraction and analysis were performed independently by two of the authors of the study (L.H., J.Z.). If necessary, the full-text article was obtained to correctly categorize the studies. The screening was followed by a thorough investigation of the full-text articles of the studies matching inclusion criteria (n = 105). In case of disagreement with regard to inclusion or exclusion of a study an agreement was established by discussion. In addition to the above-mentioned search strategy, references of studies were included and available review articles were screened for relevant articles that were left out by the search process (for detailed information on the pathway used, inclusion and exclusion criteria, confer Fig 1).

### Data acquisition

105 studies were scrutinized and information was gathered in an Excel sheet. The reviewers noted relevant information such as authors’ names, year of publication, level of evidence (according to the Oxford Centre for Evidence-based Medicine), number of patients initially included and number of patients at final follow-up and time period of follow-up. Study quality was evaluated by the Coleman Methodology Score.[23] Patients’ characteristics and epidemiologic data was worked out (mean age at the time of the accident and sex). Fractures were investigated. Classifications, mechanisms of injury and the relation of metaphyseal to epiphyseal fractures were recorded. Special focus was placed on treatment options and treatment characteristics, along with their parameters of outcome (functional, radiological outcome, shortening of the humerus) and their complications.

Relevant complications were defined as follows:

- any adverse event that resulted in a change of the initially planned treatment option or revision
- loss of reduction,
- infection*,
- implant perforations*,
- complex hardware removal*,
- fracture of material*,
- loosening of implants*,
- (temporary) elbow stiffness,
- enlarged scars*
- neurological sequelae.

Complications marked with a * were not relevant for conservative treatment options. If a fracture was initially treated conservatively and surgery was necessary due to a loss of reposition this was registered as a complication of conservative treatment. This led to an increase of patient numbers in the meta-analysis of the complication rates of conservatively treated patients in the studies concerned.[6,9]

The outcome at final follow-up was relevant for the meta-analysis. Good and excellent functional outcome was defined as good and excellent outcome according to the recommendations given by the different authors of the score. If no score was used, persistent pain, limitation of the range-of-motion larger than 20 degrees in any direction, loss of strength or any restriction in daily activities were interpreted as “moderate/poor” outcome. In addition, a subgroup analysis of severely displaced fractures (Neer III/IV, varus displacement ≥ 20°) was performed.

Any result that could be classified as “good” or “excellent” was considered as being part of the “success rate”, any other result was rated as a non-successful result (any score of “average”, “poor” or “bad”). A good or excellent radiological outcome was defined by the absence of residual angulation at final follow-up. Any arm length discrepancy in comparison to the contralateral side at final follow-up was registered. Subsequent to the meta-analysis a review of age-related factors of final outcome was performed.

The most common three different treatment options were analysed (conservative treatment, K-wires, ESIN). Differing treatment methods named in the single studies (such as plate osteosynthesis or olecranon traction) were excluded from the meta-anaylsis.

### Assessment of quality (Coleman Methodology Score)

The Coleman Methodology Score is a score consisting of 10 criteria to assess the methodology of the single studies: study size, mean follow-up, number of surgical procedures, type of study, diagnostic certainty, description of surgical procedure, postoperative rehabilitation, outcome measures, outcome assessment, and selection process.[23] The points that can be achieved range from 0 to 100 points, with 100 points being the best possible result, i.e. a study design that largely avoids the influence of chance, different biases and confounding factors.[23] The Coleman Methodology Score is an established mean to evaluate the studies’ quality.[24,25]

### Statistics

**Fig 2:**
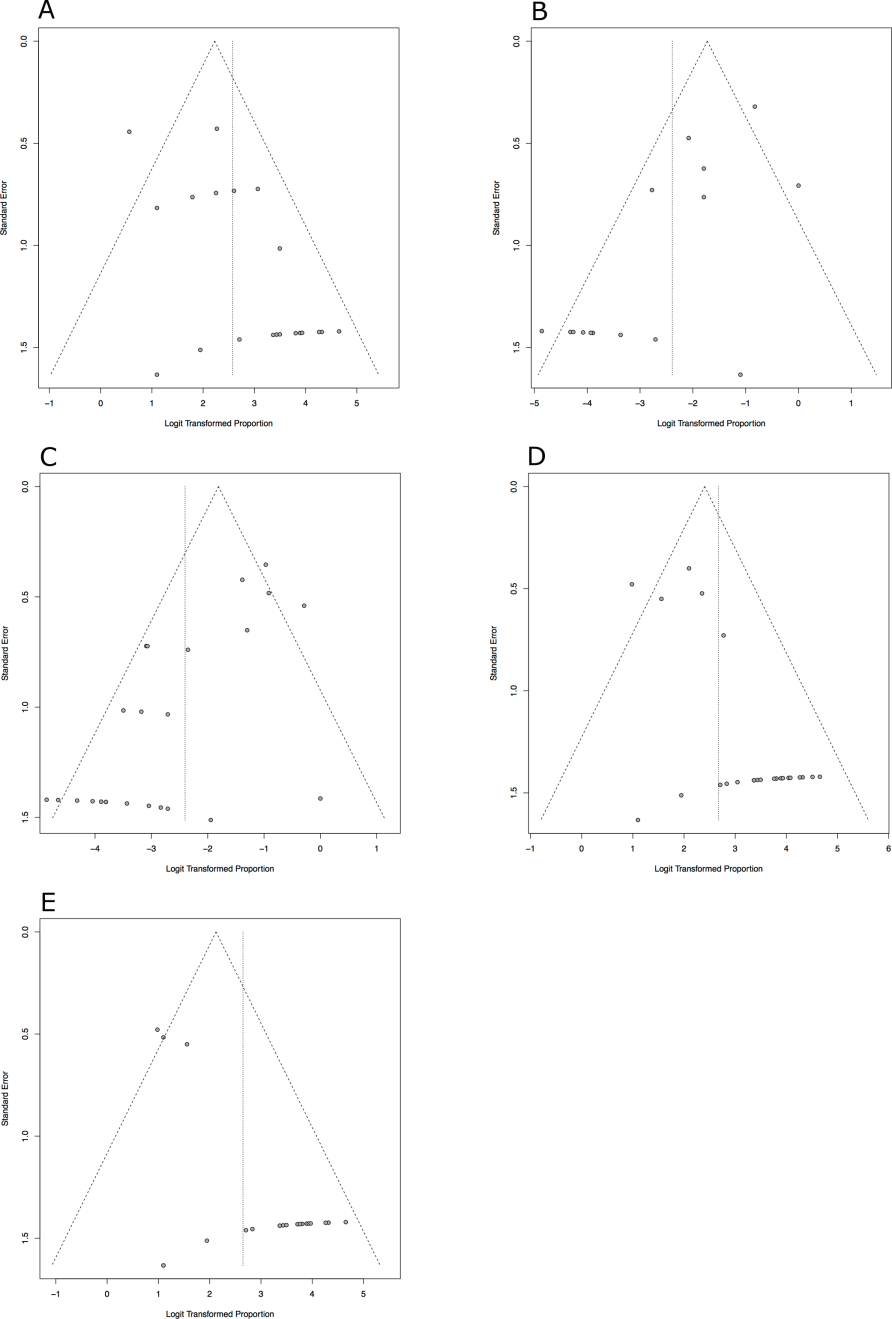
**a-e** **Funnel plots of the meta-analyses**. (a) Radiological Outcome (b) Arm length discrepancy (c) Complication rate (d) Functional Outcome (e) Subgroup analysis of severly displaced fractures. A publication bias of the five meta-analyses could not be excluded.

Statistical analysis was performed by a statistical software (Excel Version 14.4.7, IBM SPSS Version 21.0) and meta-analyses were also run by a specific software (R-Project Version 3.2.4, package ‘meta’ by G. Schwarzer). The meta-analysis was performed using the random-effects method based on the inverse variance approach. A logit transformation was applied. Heterogeneity variance was estimated with the DerSimonian-Laird estimator for tau^2.^. The Clopper-Pearson approach was used to determine the exact confidence intervals for the individual studies. Results were summarized in a chart as a forest plot. Calculation of heterogeneity was effected by the method of Higgins et al. Heterogeneity was represented by the I^2.^–value, The I^2.^ can assume values from 0 (complete consistency of the data) to 100% (complete inconsistency). A test of differences between the groups was run, a p-value of 0.05 was considered statistically significant. Funnel plots were created to evaluate the publication bias within the studies.(Fig 2 A–2E)

## Results

### Study characteristics

19 studies were analysed for the above-named parameters.[6–9,12–17,26–29] Quality assessment led to a calculation of a mean Coleman Methodology Score of 70.95 ± 7.41 points (range 57–86 points, confer Table 1). One study was of Level III evidence according to the Oxford Centre for Evidence-based Medicine, 18 studies were of Level IV evidence.

**Table 1:** Study characteristics. The studies included into the meta-analysis and their characteristics such as year of publication, mean follow-up period, treatment method described, parameters of evaluation of the functional outcome and their Coleman Methodology Score (quality assessment) are shown.

Study	Year	Sample Size	Follow-up period (mean, in months)	Treatment	Evaluation of Functional Outcome	Coleman Methodology Score
Chaus et al.[26]	2015	32	58,8	conservative, surgical	Quick DASH	62
Kraus et al.[27]	2014	31	68	ESIN, K-wire	DASH Score	72
Khan et al.[12]	2014	27	15,2	ESIN	Quick DASH	86
Canavese et al.[13]	2014	58	18,3	ESIN	Quick DASH	79
Wang et al.[14]	2014	37	24	ESIN	Neer Shoulder Score	66
Xie et al.[15]	2011	25	20,4	ESIN	Individual Evaluation (Range of Motion, Degree of Satisfaction, Return to full sports activities)	66
Bahrs et al. [28]	2009	43	39	conservative, K-wire, other methods	Constant Murley Score	77
Fernandez et al.[16]	2008	35	26	ESIN	Constant Murley Score	69
Chee et al.[17]	2006	14	14,6	ESIN	Individual Evaluation (Range of Motion, Return to full activity)	72
Schwen-denwein et al.[6]	2004	16	23,8	conservative, K-wire	Individual Score (based on Function, Pain, Subjective Satisfaction)	57
Karatosun et al. [30]	2003	7	54	conservative	Individual Evaluation (Range of Motion, Pain)	72
Dobbs et al.[7]	2003	28	48	conservative, K-wire, other methods	Individual Evaluation (Pain, Strength, Range of Motion, Participation in sports, Performance of activities)	69
Burgos-Flores et al. [8]	1993	22	81,6	K-wire	Individual Evaluation (Range of Motion, Dysmetria, Activity restriction, Pain)	74
Larsen et al.[1]	1990	64	108	conservative	Individual Evaluation (Muscle strength, Range of Motion, Subjective Discomfort)	85
Frey et al.[9]	1989	56	60	conservative, K-wire, other methods	Evaluation of function according to Razémond and Baux	63
Giebel et al.[29]	1983	23	55,2	K-wire, other methods	Individual Evaluation (Complaints, Range of Motion)	69
Dameron et al.[5]	1969	69	84	conservative	Individual Evaluation (Subjective Discomfort/Restriction, Atrophy, Range of Motion, Strength)	65
Neer et al.[3]	1965	89	57,6	conservative, other methods	Individual Evaluation (functional recovery)	70
Nilsson & Svartholm [31]	1965	44	93.6	conservative	Individual Evaluation (Discomfort, Range of Motion, Strength, Atrophy)	70

### Characteristics of the patient population

A total of 735 patients were included, 643 were available for a follow-up examination (overall follow-up rate 87.5%) that took place at an average of 48.2 months (range 14.6 to 108 months) after the initial treatment. The patients included had a mean age of 11.8 years (range 8.5 to 14.5 years) and a ratio of male to female patients of 1.4:1.

### Fracture characteristics and cause of injury

408 fractures were epiphyseal fractures (predominantly Salter-Harris type II), and 193 fractures were metaphyseal fractures. Some of the studies did not indicate the ratio of epi- to metaphyseal fractures and could not be included into the count. The most common mechanism leading to those fractures of the proximal humerus were direct traumata to the proximal humerus (37.5%), followed by sports- and physical activity-related accidents (30.49%), or falls (12.6%). Road accidents (6.97%) were rarely responsible for the occurrence of those fractures.

### Options of treatment

Conservative treatment consisted of immobilisation in a sling, Velpeau bandage, Desault bandage, cast or hanging cast for a variable period of time (mean time 3.69 weeks, range 1 to 18 weeks) depending on grade of displacement and children’s age. Sometimes closed or open reduction was performed before immobilisation. Karatosun et al. used a special two-prong splint for severely displaced fractures (Neer grade 3 to 4) which was removed after 2 to 3 weeks. They had excellent results without any complications. K-wire treatment was performed in a percutaneous or open technique with the use of two or three K-wires. A post-operative immobilisation was applied for 3.8 weeks (range 3.2 to 6 weeks). ESIN was performed in a retrograde technique using one or two nails. Post-operatively the shoulder was immobilised in a sling or cast for an average of 1.9 weeks (range 2 days to 3 weeks).

### Radiological outcome

**Fig 3:**
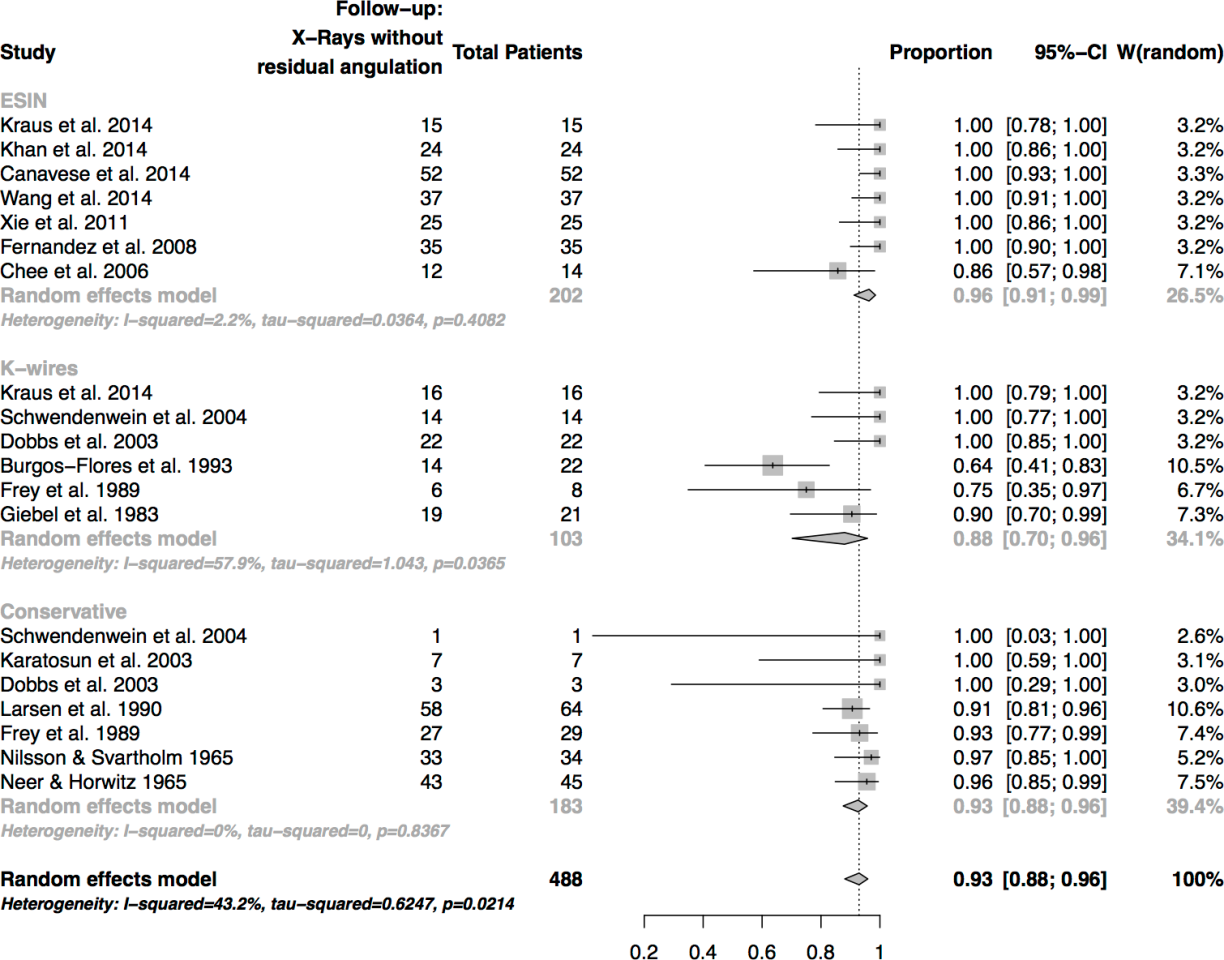
Analysis of X-Rays at final follow-up. Only X-rays without residual deformity were weighted as a successful treatment.

Good to excellent outcome was achieved in 93% of the cases. (Fig 3) The success rate was higher in patients treated by ESIN (96%) than in those treated conservatively (93%) or in patients treated by K-wires (88%). Differences were not statistically significant (p-value = 0.21)

### Arm length discrepancy (shortening)

**Fig 4:**
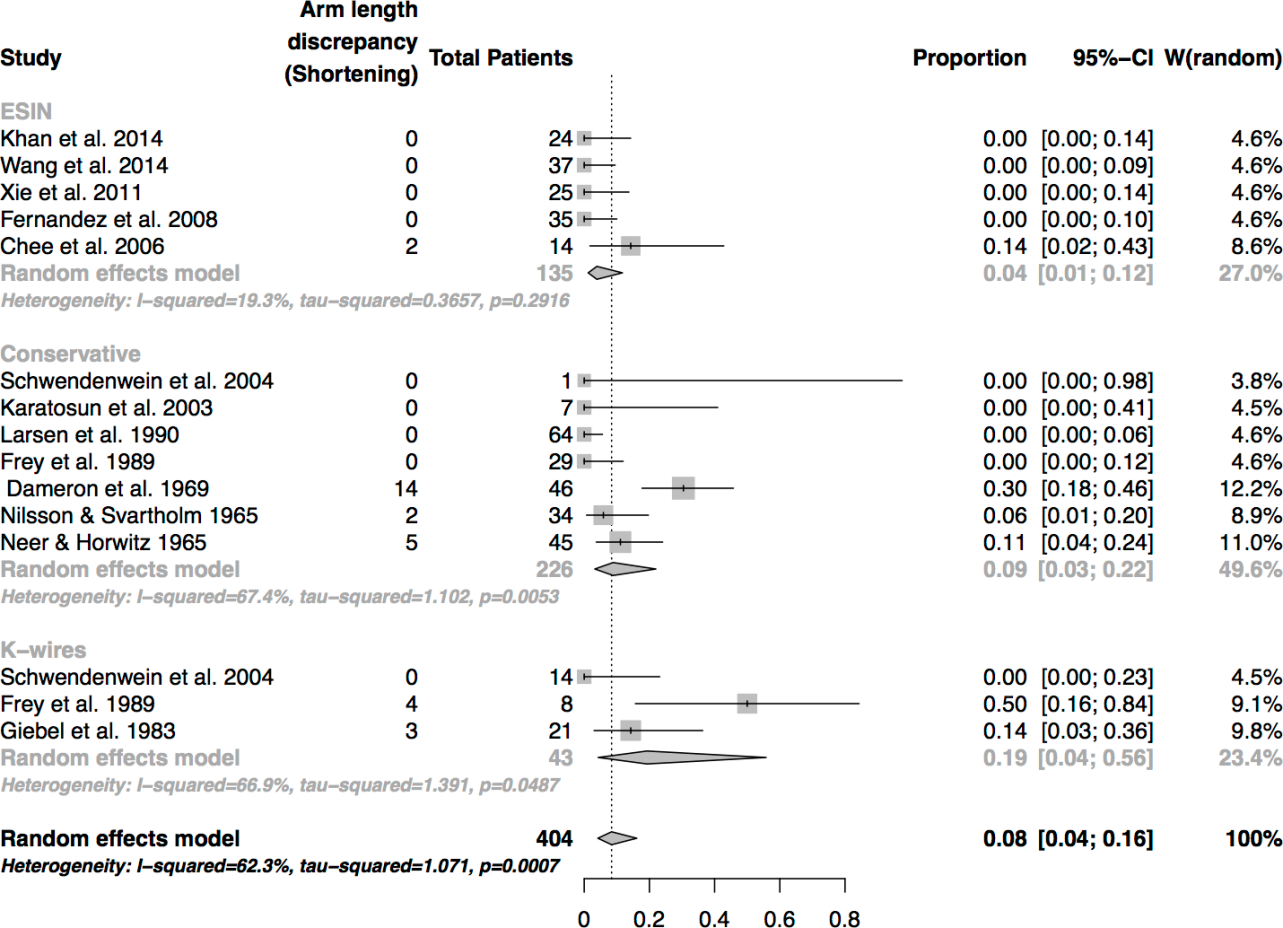
Analysis of arm length discrepancies at final follow-up.

The rate of humeral shortening in the course of healing was particularly high in patients treated by K-wire osteosynthesis (19%), followed by conservative treatment (9%) and osteosynthesis by ESIN (4%) (Fig 4). The difference between the groups was not of statistical significance (p = 0.22).

#### Complications

**Fig 5:**
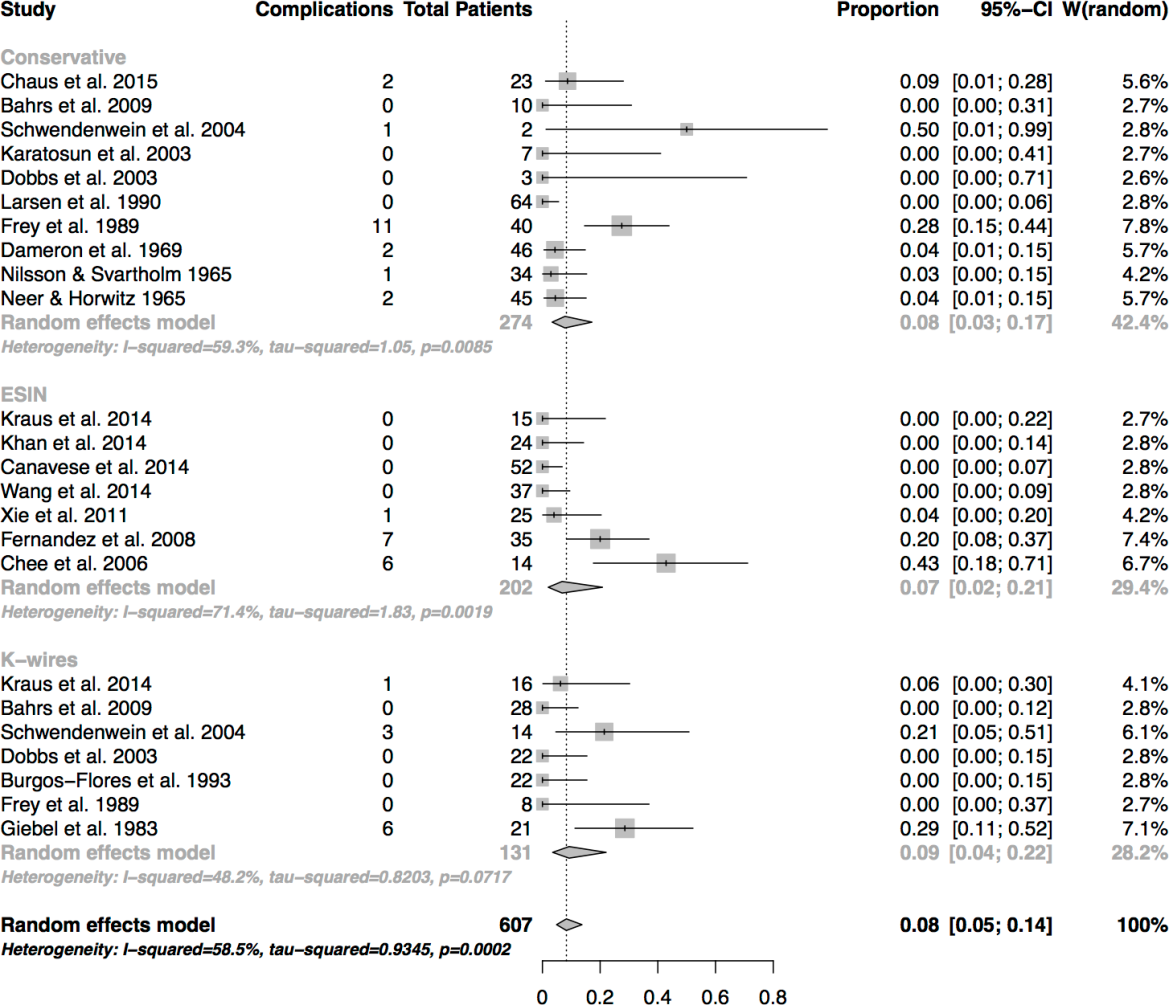
Analysis of complications. Registration of complications occurring during the healing process.

There was a complication rate of 8% in all patients treated conservatively. The complication rate was lower after treatment by ESIN (7%) than by K-wires (9%) (Fig 5). Differences in complication rates did not differ to a significant extent (p = 0.92)

#### Functional outcome

**Fig 6:**
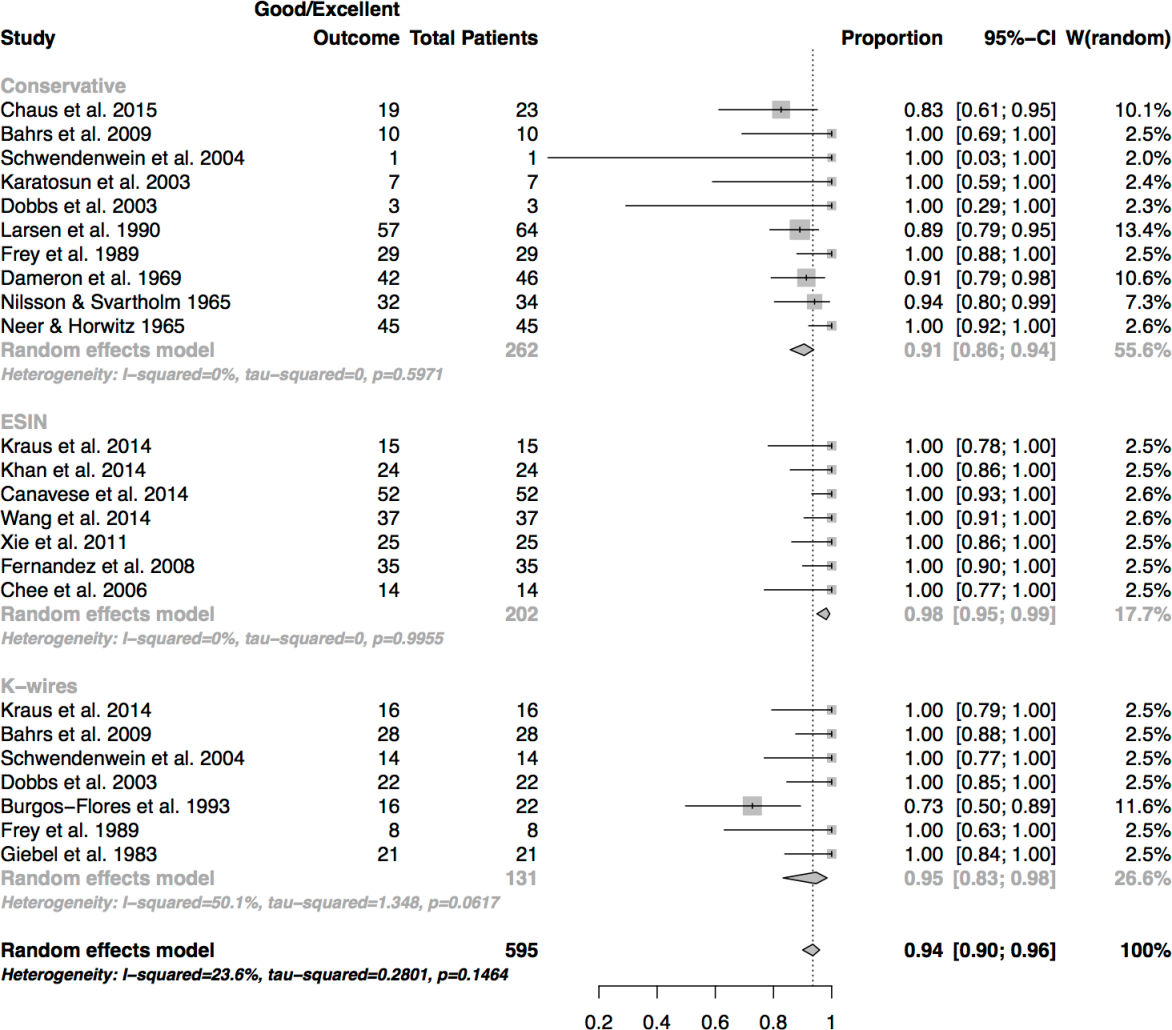
Analysis of success rates of functional outcome (rate of good and excellent outcome).

Concerning good to excellent functional outcome there were statistically significant differences between the treatment methods. (Fig 6) The best results were recorded in the group of patients treated by ESIN (98%), followed by osteosynthesis by K-wires (95%) and conservative treatment (91%). The results reached statistical significance (p = 0.01), with the rates of ESIN and K-wire fixation being significantly higher than conservative treatment.

### Functional outcome of severely displaced fractures

**Fig 7:**
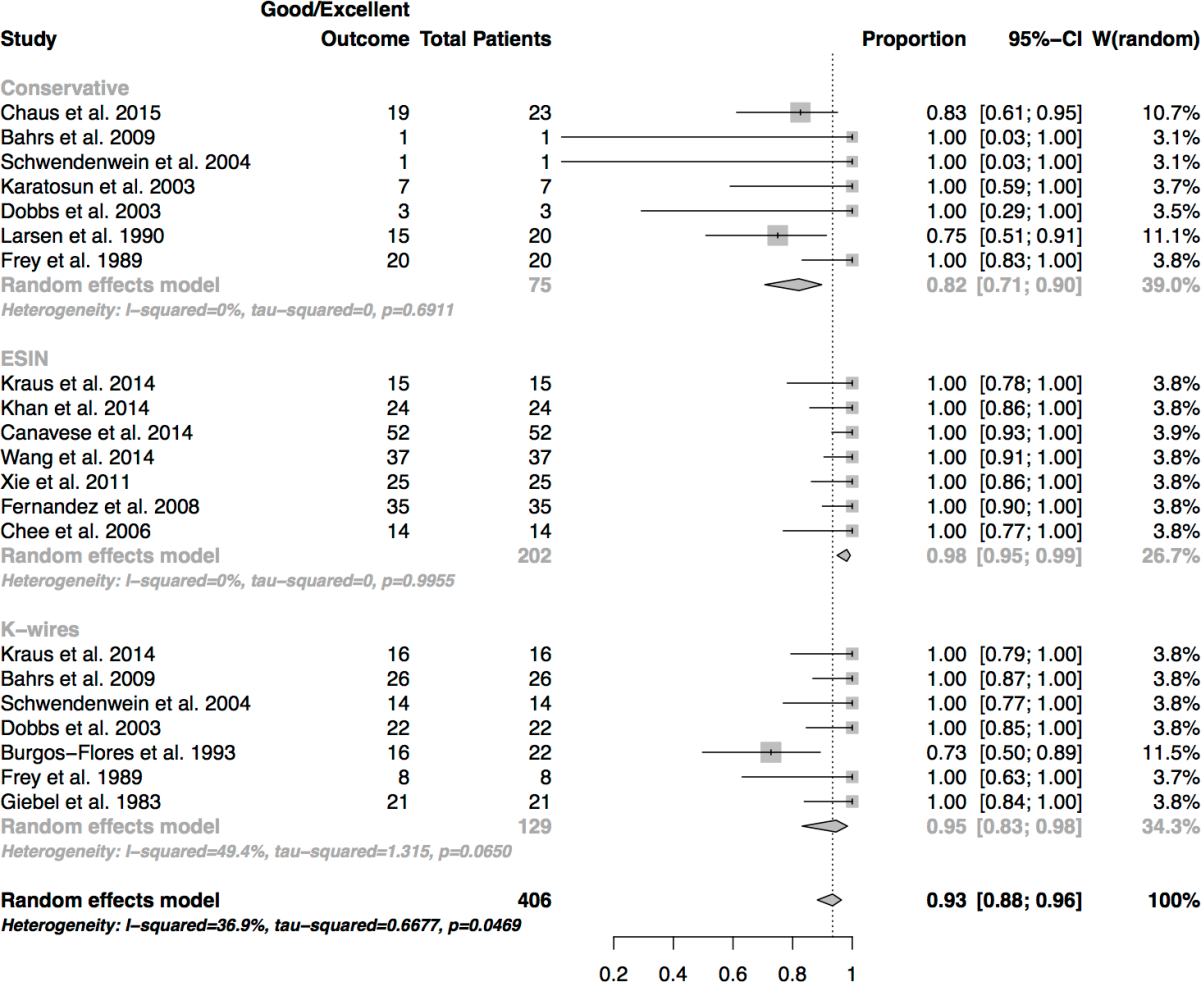
Sub-group analysis of severely displaced fractures. Success rates of functional outcome.

In comparison to the overall functional outcome results the subgroup analysis highlighted differences between conservative and surgical treatment. (Fig 7) The best results were seen in the patients treated by ESIN (success rate: 98%), which were higher than in the patients treated by K-wire osteosynthesis (success rate: 95%) and in conservatively treated patients who only showed good and excellent results in 82% of the displaced fractures. ESIN and K-wire fixation had significantly higher success rates than conservative treatment (p < 0.001)

### Outcome factors by age

The studies that were included into the meta-analysis were searched for age-related factors that had a negative effect on the final functional outcome.

#### Conservative treatment

Generally speaking, the final outcome seems to worsen by age if conservative treatment options are chosen. The age limits set differ between the single studies. For non- or slightly displaced fractures, conservative treatment can be applied safely independent of the patient’s age. [1,3,28]Until the age of 10, conservative treatment is a safe treatment option, even for severely displaced fractures: Larsen et al.[1] reported in their large series of conservatively treated patients that a complete remodelling was only observed in patients under the age of 8 years, even in fractures of Neer grade IV. The children with the widest angulation at follow-up were at least 11 years of age and all of them had a Neer grade IV fracture. In the study of Neer et al. no instance of persistent arm length discrepancies was noted in a child under 11 years of age.[3] An increase of residual deformities at final follow up seems to occur from the age of 12 years onwards.[31] From the age of 13 onwards conservative treatment should be avoided: Chaus et al. reported that a one-year increase in age was related with an increase of the odds of a less than desirable outcome by a factor of 3.81. All of the conservatively treated patients with a less than desirable outcome were at least 13 years of age.[26] Dameron et al. reported patients who had limitations in their final range-of-motion; all of them were 14 years and older.[5]

An exception of this rule were the results of Karatosun et al.[30] They introduced a custom-made cast and had a range of patient age of 11.25 to 15.67 years. All of the patients had a near-normal range of motion and excellent strength at final follow-up.

#### Osteosynthesis by K-wires

In the study of Kraus et al. all of the patients treated by K-wires were 11 years and older. All of them had favourable functional results.[27] The same result was reported by Giebel et al.; the range of age in their study was 13 to 16 years.[29] All of the patients had a good to excellent function at the final follow-up. Schwendenwein et al. reported of 7 patients with the age of 10 years and younger and of 7 patients with the age of more than 10 years of age who were treated by K-wire osteosynthesis.[6] All of them had a good to very good outcome without decrease in mobility. Burgos-Flores et al. observed 6 patients in their cohort who didn’t have a good or excellent outcome.[8] All of them were 10 years of age or older.

Bahrs et al. proved the K-wires in their study to be a safe treatment option for displaced fractures both in patients of less than 10 years of age and in patients of more than 10 years of age.[28]

#### Osteosynthesis by ESIN

In several studies reporting outcomes after the treatment by ESIN patient cohorts were separated age-independently into patients who were 10 years of age or younger[12,14,15,32], respectively 11 years of age or younger.[13] Some of the studies separately regarded the patients of more than 13 years of age.[14,16] Chee et al. had a range of age of 12 to 15 years in their study.[17] All of the patients showed good to excellent functional results irrespective of their age. Khan et al. found no difference between the patients of less and of more than 10 years of age.[12]

### Summary–development of a treatment algorithm

**Fig 8:**
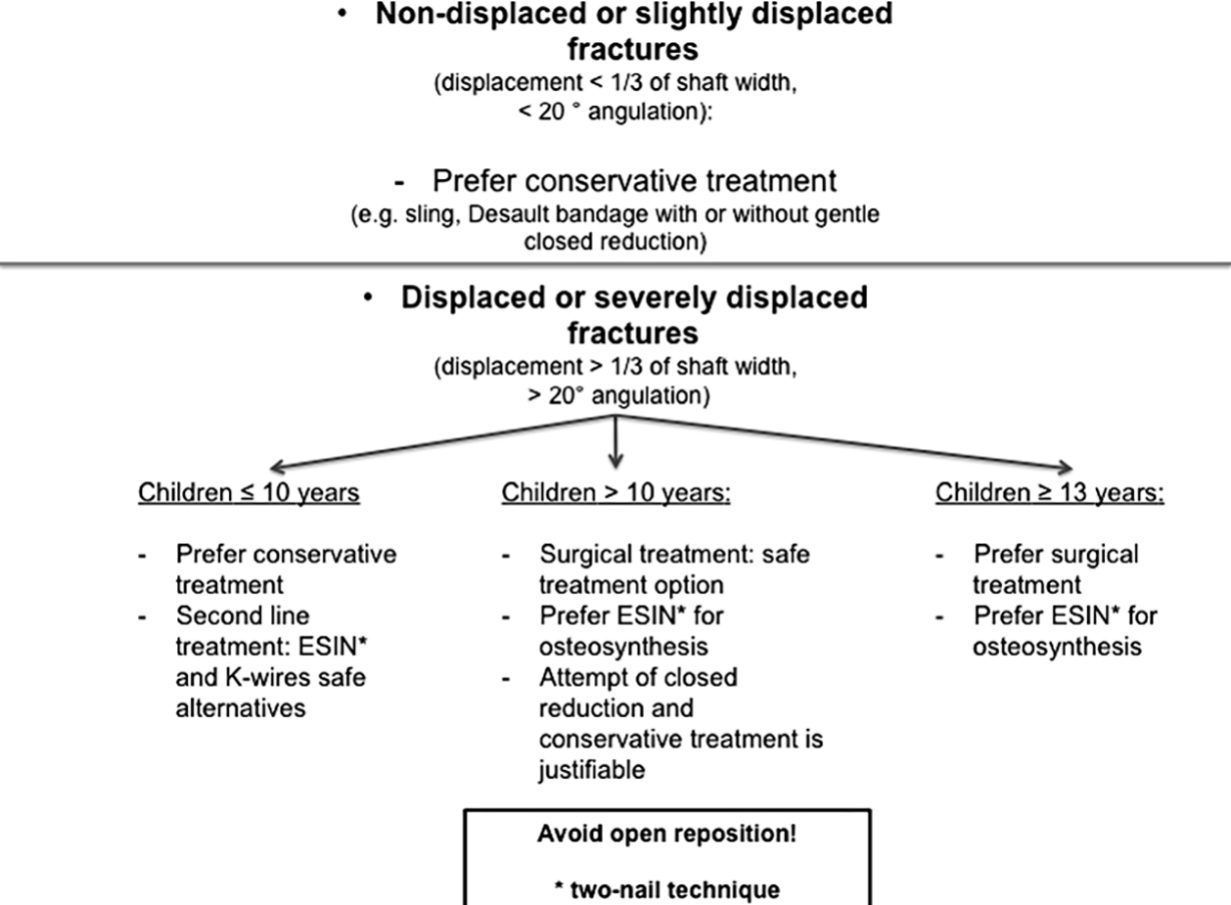
Algorithm for the treatment of proximal humeral fractures in children and adolescents.

For the development of the algorithm the results of the meta-analysis (treatment options, treatment according to the fracture severity) were combined with the age-specific findings of the systematic review. All of the studies were additionally screened for results of open versus closed reduction techniques. The information was gathered and a decision tree was created based on these facts (Fig 8). Details are discussed in the section “discussion” below.

## Discussion

The aim of this review and meta-analysis was to find an answer to the question in which cases of proximal humeral fractures in children and adolescents a conservative treatment should be applied and in which cases a surgical treatment should be preferred.

Proximal humeral fractures are rarely seen in this patient cohort. Thus, clinical studies treating the subject usually comprise a low patient number and are usually of a retrospective nature. Many of the studies have only a short period of follow-up and low follow-up rates. Treatment methods are usually reported without comparison to an alternative method and in many of those studies evaluating different methods outcome parameters were not reported separately for the different treatment options.

Therefore, conclusions in this meta-analysis and review are only based on the best available data: only studies with a follow-up of more than 12 months and with a follow-up rate of more than 65% of the patients were evaluated in order to ensure the highest possible quality of advice.

### Radiological outcome

None of the studies included could reveal a direct correlation between persistent axial deviations subsequent to a fracture of the proximal humerus and an adverse clinical outcome. In short- to mid-term follow-up examinations a residual angulation did not belong to the factors leading to an unfavourable clinical outcome.[31] Larsen et al. had the longest median period of time from fracture to final follow-up examination (9 years).[1] In accordance with the results of Nilsson et al. (mean follow-up 7.8 years) they observed complete correction of residual angulation even in cases of severely displaced fractures until the age 8–9 years.[1,31]. An extraordinary capacity of remodelling of significantly displaced fractures in young children (mean age 7.5 years) was confirmed.[9] Another study found out that until the age of 11 years, a remodelling of residual angulation up to 20° could be observed.[5] Larsen et al.[1] found the greatest residual angulation in children above the age of 11 years with an initial fracture displacement of more than 2/3 if the shaft width (most severe type of fracture), Nilsson et al.[31] most frequently found residual angulation at an average age of 12 years and even the only patient with considerable displacement was symptom-free. The explanation provided for the discrepancies between radiological aspect and function is the wide range of motion of the glenohumeral joint compensating for limitations.[1,31] Unfortunately, there are no studies providing long-term results of those fractures on the glenohumeral joint. Thus, it remains unclear whether these compensation mechanisms of the shoulder promote degenerative articular changes in the future. Surprisingly the highest rate of persistent angulation on X-rays was seen in K-wire osteosynthesis. Burgos-Flores et al. observed 8 patients with residual angulation. Six of them had a reduced mobility and dysmetria at follow-up, though only 2 of them were aware of the loss in range-of-motion in everyday activities.[8] All of those patients were 13 years or older.

### Arm length discrepancies

In the studies included there were no cases of growth enhancement after proximal humerus fractures in children. The highest rate of arm length shortening was seen in the course of osteosynthesis by K-wires. Frey et al.[9] observed a rate of 50%. Nonetheless, all of the patients concerned had the most severe displacement of more than ½ of the humeral shaft width, and 75% of the fractures required an open reduction. Giebel et al. reported 3 (of 21) cases of shortening, and the patients concerned were 11 and 17 (n = 2) of age.[29] In their patient cohort only fractures, which required reposition and which were instable after reposition were treated by K-wire osteosynthesis. In the conservatively treated patient cohort, however, humeral shortening was rarely observed. Larsen et al. (20 fractures of more than 1/3 displacement of shaft width, mean patient age ≤ 15 years) as well as Frey at al. (predominantly young children, average age 7.5 and 8.8. years) did not find any case of humeral shortening in their large series of conservatively treated patients.[1,9] Neer et al.[3] registered an increasing incidence of arm length shortenings with age (> 11 years) even after fractures with little displacement (Neer grade I or II). Dameron et al.[5] had the highest rate of arm length discrepancies in their series. All but one of those fractures had a significant initial displacement and required a manipulative reduction under general anaesthesia.[5] In fractures treated by ESIN humeral shortening was the exception and was only seen in the earliest study where a one-nail technique for severely displaced fractures was used.[17]

### Complications

Conservative treatment had low complication rates and the complications observed frequently showed loss of reduction.[6,9,26] Nilsson et al.[31] reported one case of occasional numbness and muscular atrophy after immobilization in a supporting bandage. A closer look at the case revealed that displacement was so severe that an open reduction had to be performed before immobilisation. The same problem was found in the study of Neer et al. who reported that 8 of their patients had open reduction prior to the immobilisation. Four of them lost their reposition and returned to the original deformity.[3] The complication rate after open reduction made the authors turn away from this procedure. The complications observed by Dameron et. al.[5] were temporary neurological symptoms. One patient required K-wire traction previous to the immobilisation and another patient showed temporary symptoms of a brachial plexus paresis after an overhead-immobilisation in spica cast.

Osteosynthesis by K-wires showed a complication rate of 9 percent. The high rate of complications in two studies was due to 6 cases of unsightly hypertrophic scars after open reduction and osteosynthesis.[6,29] The remaining complication was a superficial skin irritation leading to an early removal of K-wires.[27] The elevated risk of complications after open reduction should therefore entail an avoidance of open reposition.

At first glance, the complication rate after osteosynthesis by ESIN seems to be high. If one takes a closer look, the main part of those complications appeared in the earliest published studies[16,17], with a preference of a one-nail technique[17], respectively with a one-nail technique and switch to a two-nail technique in the course of “experience-gaining”.[16] Complications such as temporary elbow stiffness were resolved by improvement of the operation technique (shortening of the nails at the distal end at 2 cm instead of > 3 cm).[17] Other complications in those early studies were: perforation of the nail (n = 2), loss of position (n = 2 one-nail technique, n = 1 two-nail technique), nail–misplacement and a postoperative hematoma with need for early revision or a difficult implant removal in 2 cases.

Since 2011 five studies have been published and were included in our meta-analysis with 153 patients treated by ESIN for significantly displaced fractures of the proximal humerus by a two-nail technique. Since then, only one complication has been registered (early revision due to protrusion of a nail through the skin).[15]

### Functional outcome

First, an analysis of the overall functional outcome was performed leading to a success rate of > 90% irrespective of which treatment method was chosen.

Neer et al. in their study of proximal humerus fractures in children and adolescents made a thorough investigation of the characteristics of those fractures and their findings are still the basis of up-to-date knowledge.[3] They came to the conclusion that lesions with grade I and II—displacement should be treated without reduction by simple immobilization in a sling and swathe which generally resulted in a favourable functional outcome.[3] This was confirmed in other studies, where sometimes a gentle closed reduction was added.[1,5,7,28,31] The question which is being debated is, which treatment should be chosen for the significantly displaced fractures. This is backed up by the fact that the studies which have been published since 2010 have only treated this subject.[12–15,26,27] Since 2000 only two studies meeting the inclusion criteria comprising more than 3 patients have been published which evaluated conservative treatment options for significantly displaced fractures.[26,30]

This was the reason why a subgroup analysis of significantly displaced fractures was performed in the present study. The definition of a displaced fracture was defined by an angular displacement of more than 20 degrees in accordance with findings in other studies [5,9] and by a shaft displacement of Neer grade III and IV. The results of this subgroup analysis were striking. Conservative treatment options fell behind operative treatment options (ESIN and osteosynthesis by K-wires) with a success rate of 82% against a rate of 95% (K-wires) and 98% (ESIN). In addition, Frey et al. did not report any moderate or poor outcome subsequent to conservative treatment (Desault, reposition and Desault). Nonetheless, 11 displaced fractures which underwent an initial fracture reposition had to be treated secondarily by extension or surgery, as the immobilisation in a Desault bandage could not retain the fragments (sub-summarized in Fig 4).[9] It is therefore highly probable that displaced fractures which require initial reposition are not a good indication for conservative treatment, especially in older patients with less remodelling potential.

Karatosun et al.[30] presented a custom-made elaborate cast which showed very good results in the treatment of displaced fractures.

Limitations of this meta-analysis and review naturally correspond to the limitations of the single studies included, such as study design and the use of shoulder scores as outcome parameters, which are not validated for children and adolescents.[27] A publication bias could not be excluded which is due to the small study size (small study sizes show large effects.). Since proximal humerus fractures are rare fractures of children, studies usually were of a retrospective nature (18/19 studies). A high quality was ensured by choosing a period of follow-up ≥ 12 months and a follow-up rate ≥ 65% as inclusion criteria.

The algorithm as such is structured to serve as a guide for an evidence-based management of proximal humerus fractures in children. In summary, based on the data of highest quality, non- or slightly displaced fractures (angulation < 20° or Neer grade I/II) can safely be treated without a surgical intervention–independent of the patient’s age. Displaced or severely displaced fractures can safely be treated by surgical intervention (preferably by ESIN) as it usually leads to an excellent clinical outcome–independent of the patient’s age. Nonetheless, especially in patients until the age of 10 years a surgical intervention might be an over-treatment due to the substantial remodelling capability of the proximal humeral epiphysis. Until the age of 8 years, even severely displaced fractures will undergo a complete remodelling process. The remodelling capability decreases with age and varies between the individuals. [1,3] In the age group of 10 to 13 years conservative treatment of displaced fractures increasingly leads to arm length discrepancies and residual angulation. Decisions should be made in a case-to-case decision, based on the current level of development or rather on the skeletal maturity of the child. From the age of 13 years onwards, conservative treatment of displaced fractures leads to a high risk of impaired functional outcome and is not recommended.

Open reduction led to higher complication rates and should be avoided whenever possible.

## Conclusion

The objectives of this review and meta-analysis were to accurately search for tangible factors of favourable and unfavourable outcome of proximal humerus fractures in children and adolescents. The algorithm was based on these data (comprising functional and radiological outcome parameters, risk factors for arm length discrepancies and complications).

The preference of ESIN over K-wires is due to the excellent functional outcome, the low complication rates of the two-nail technique and the possibility of early mobilisation (immobilisation only necessary immediately postoperatively for pain relief). The preference of conservative treatment over surgical treatment in 10 year-old children or younger is due to the above-mentioned proof of excellent remodelling capacities in this patient age. Therefore, the risks of an operation can be avoided without having to counterweigh the risk of residual deformity and a possible unfavourable functional outcome. From the age of 11 years onwards the risk of residual deformity increases. From the age of 13 years onwards an increase of undesirable functional outcomes can be observed. Thus, conservative treatment should be avoided for significantly displaced fractures in this patient age.

## Supporting information

S1 File PRISMA checklist. (PDF)

S2 File PRISMA flow diagram. (PDF)
