# Supplementary material for: Age- and severity-adjusted treatment of proximal humerus fractures in children and adolescents—A systematical review and meta-analysis
Source: PLoS One. 2017 Aug 24;12(8):e0183157. doi: 10.1371/journal.pone.0183157 (PMC5570290; doi:10.1371/journal.pone.0183157)
Supplement: S2 File — (PDF) [file pone.0183157.s002.pdf]

PRISMA Flow Diagram

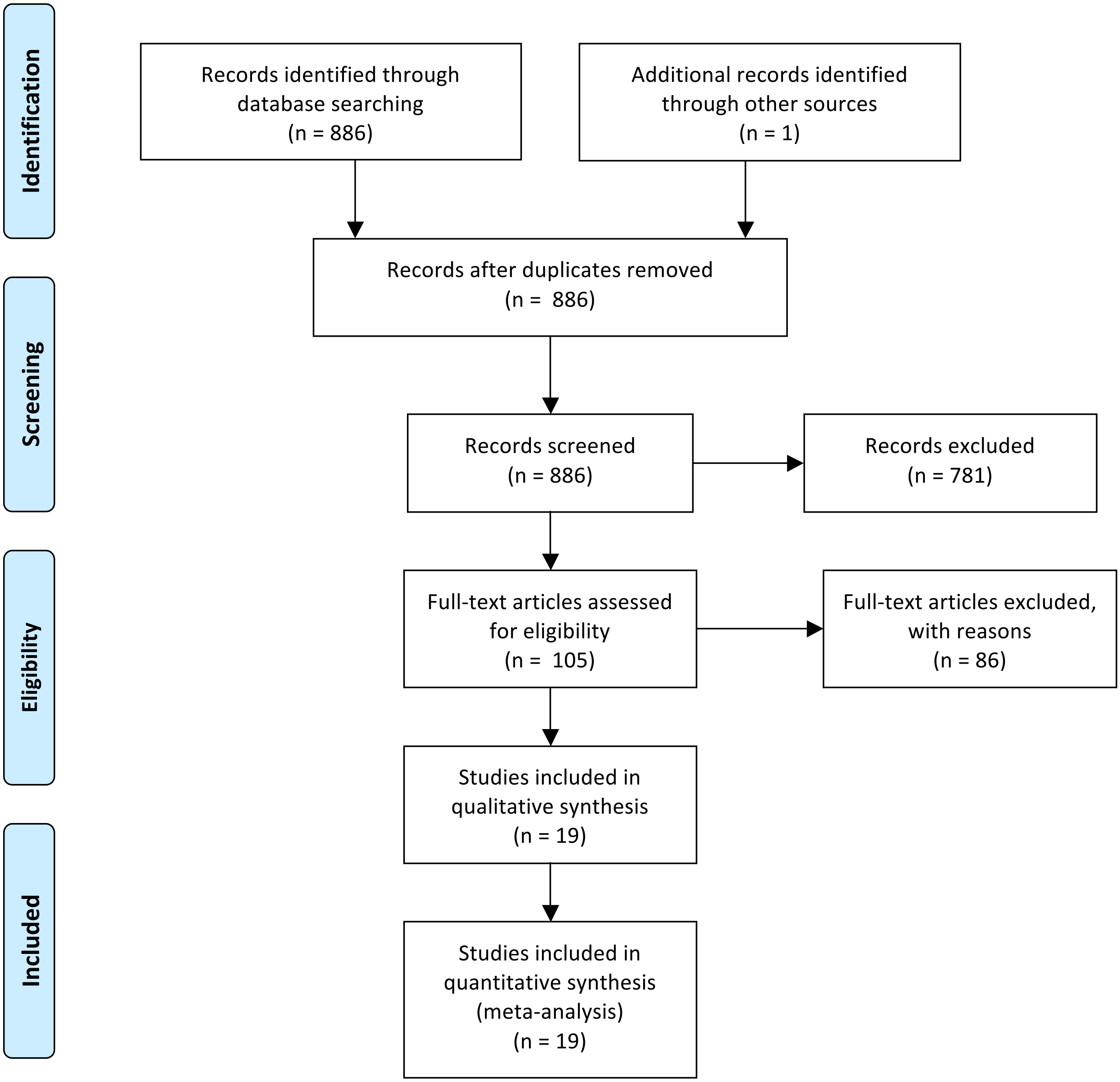

From: Moher D, Liberati A, Tetzlaff J, Altman DG, The PRISMA Group (2009). *P*referred *R*eporting *I*tems for *S*ystematic Reviews and *M*eta-*A*nalyses: The PRISMA Statement. PLoS Med 6(7): e1000097. doi:10.1371/journal.pmed1000097

For more information, visit www.prisma-statement.org.
